# EGFR-targeted nonviral NIS gene transfer for bioimaging and therapy of disseminated colon cancer metastases

**DOI:** 10.18632/oncotarget.21028

**Published:** 2017-09-16

**Authors:** Sarah Urnauer, Andrea M. Müller, Christina Schug, Kathrin A. Schmohl, Mariella Tutter, Nathalie Schwenk, Wolfgang Rödl, Stephan Morys, Michael Ingrisch, Jens Bertram, Peter Bartenstein, Dirk-André Clevert, Ernst Wagner, Christine Spitzweg

**Affiliations:** ^1^ Department of Internal Medicine IV, University Hospital of Munich, LMU Munich, Munich, Germany; ^2^ Department of Pharmacy, Center of Drug Research, Pharmaceutical Biotechnology, LMU Munich, Munich, Germany; ^3^ Department of Clinical Radiology, University Hospital of Munich, LMU Munich, Munich, Germany; ^4^ Department of Nuclear Medicine, Radiopharmacy, Klinikum rechts der Isar der Technischen Universität München, Munich, Germany; ^5^ Department of Nuclear Medicine, University Hospital of Munich, LMU Munich, Munich, Germany

**Keywords:** sodium iodide symporter, gene therapy, theranostic application, nonviral EGFR-targeted gene transfer, colon cancer metastases

## Abstract

Liver metastases present a serious problem in the therapy of advanced colorectal cancer (CRC), as more than 20% of patients have distant metastases at the time of diagnosis with less than 5% being cured. Consequently, new therapeutic approaches are of major need together with high-resolution imaging methods that allow highly specific detection of small metastases.

The unique combination of reporter and therapy gene function of the sodium iodide symporter (NIS) may represent a promising theranostic strategy for CRC liver metastases allowing non-invasive imaging of functional NIS expression and therapeutic application of ^131^I.

For targeted NIS gene transfer polymers containing linear polyethylenimine (LPEI), polyethylene glycol (PEG) and the epidermal growth factor receptor (EGFR)-specific ligand GE11 were complexed with human NIS DNA (LPEI-PEG-GE11/NIS). Tumor specificity and transduction efficiency were examined in high EGFR-expressing LS174T metastases by non-invasive imaging using ^18^F-tetrafluoroborate (^18^F-TFB) as novel NIS PET tracer. Mice that were injected with LPEI-PEG-GE11/NIS 48 h before ^18^F-TFB application showed high tumoral levels (4.8±0.6% of injected dose) of NIS-mediated radionuclide uptake in comparison to low levels detected in mice that received untargeted control polyplexes. Three cycles of intravenous injection of EGFR-targeted NIS polyplexes followed by therapeutic application of 55.5 MBq ^131^I resulted in marked delay in metastases spread, which was associated with improved animal survival.

In conclusion, these preclinical data confirm the enormous potential of EGFR-targeted synthetic polymers for systemic NIS gene delivery in an advanced multifocal CRC liver metastases model and open the exciting prospect of NIS-mediated radionuclide therapy in metastatic disease.

## INTRODUCTION

Metastatic dissemination of tumor cells is mostly responsible for the high mortality of patients with colorectal cancer (CRC), especially due to late diagnosis. Although the incidence of CRC is decreasing, CRC is the third leading cause of cancer-related death worldwide [1–3]. For early stages, surgical resection represents the first-line therapy concept, while in advanced stages a combination of surgical resection and chemotherapy are applied. As the 5-year survival of patients at the metastatic stage is about 6%, new potent therapeutic options are highly important [4].

For evaluation of novel CRC therapy approaches, especially in metastatic disease, it is a prerequisite to have models that give a detailed reflection of the morphological and molecular situation of cancer. Therefore, the establishment of advanced preclinical models that mimic human metastases, tumor microenvironment and tumor stroma are indispensable to obtain a valuable prediction of the therapeutic efficacy of new treatment strategies for a future application in humans [5, 6]. To this end, a human colon carcinoma metastases model was established by injecting CRC cells directly into the spleen. This resulted in induction of multifocal liver metastases highly similar to the metastatic process in humans, where the liver represents the most common metastatic site in patients with CRC [7].

A new evolving sector in cancer treatment is based on gene therapy [8], as utilizing and manipulating the particular biological characteristics of tumor cells provide a wide range of possible interactions to target different steps of the carcinogenesis process. In recent years there have been many attempts to knock out genes that are involved in tumor formation, growth and dissemination, to introduce suicide genes as well as inserting new genes containing specific anti-tumoral functions [9]. In this regard, the sodium iodide symporter (NIS) represents an advantageous and highly efficient target gene, as functional NIS gene expression allows exact localization of the tumorous tissue by administration of NIS specific tracers such as ^123^I, ^124^I and the novel NIS positron-emissions-tomography (PET) tracer ^18^F-tetrafluoroborate (^18^F-TFB) and at the same time gives the possibility of cytotoxic radioiodide treatment.

This dual concept, which is based on the iodide uptake activity of NIS [10], was proven by several groups including our own using a broad spectrum of gene delivery vehicles, such as viral vectors, mesenchymal stem cells and non viral polymer-based vectors [3, 11–25]. Especially non-viral polymeric vehicles proved to be advantageous both technically and biologically due to the ease of adjusting chemical properties of synthetic particles for increased efficacy and biocompatibility [26, 27]. A modular synthesis concept allows incorporation of functional groups, such as polyethylene glycol (PEG) to achieve shielding against blood components *in vivo* and polymers can specifically be designed to interact with individual tumor tissue properties to achieve tumor specific targeting [28]. To this end, polymers were coupled to ligands that bind to receptors overexpressed on the tumor cell, which results in tumor-directed uptake of delivery vehicles and minimizes off-target effects. In former studies, we tested polyplexes with an EGFR-specific targeting peptide (GE11) as NIS gene delivery vehicles in a subcutaneous HuH7 xenograft tumor model [15] as well as in orthotopic pancreatic ductal adenocarcinoma [29], which resulted in high tumor-specific NIS-mediated iodide accumulation.

To take the next step in the translation of this novel theranostic NIS gene strategy from laboratory scale to the clinical situation, in the current paper, we applied the NIS pDNA polyplexes in a high EGFR-expressing tumor model of metastatic colorectal cancer. This setting mimics the complex and functional environment of metastases including tumor stroma and tumor microenvironment and serves as ideal model for evaluation of the feasibility of the NIS gene therapy concept after EGFR-targeted nonviral gene transfer. In addition, the combination with the novel NIS PET tracer ^18^F-TFB as improved diagnostic tool is utilized for optimized localization of single metastases and the therapeutic potency after application of ^131^I is evaluated.

## RESULTS

### NIS-mediated iodide uptake studies *in vitro*

**Figure 1:**
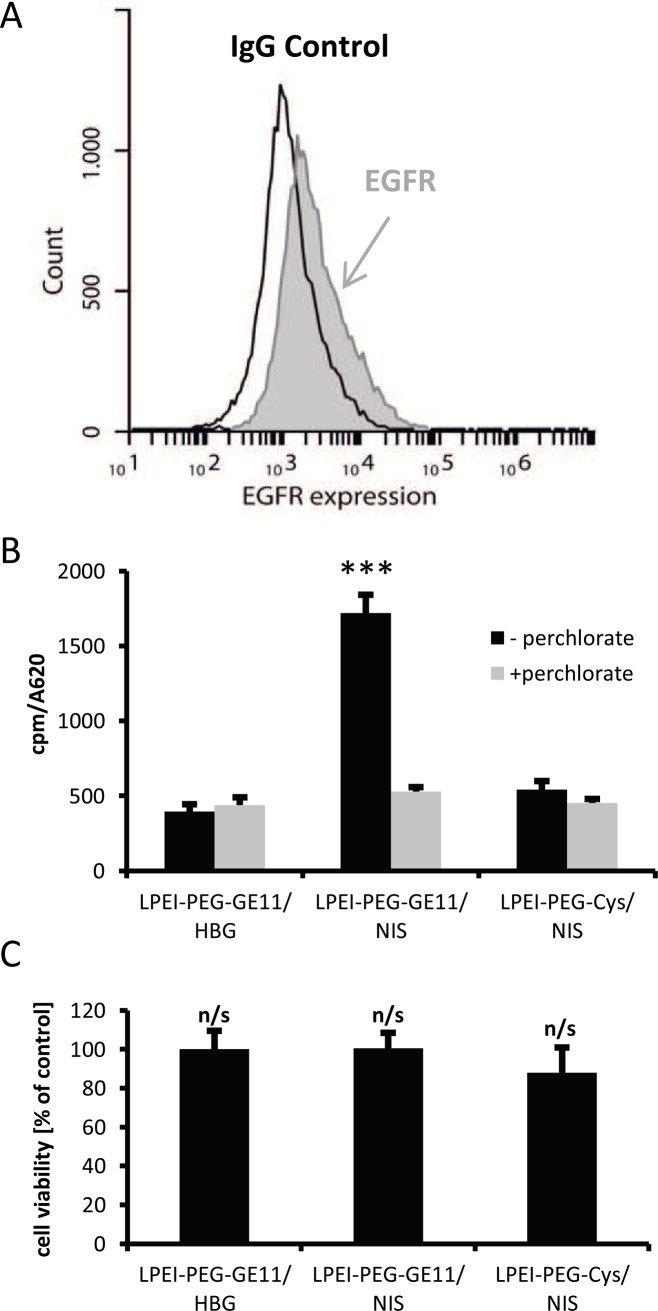
NIS-mediated ^125^I uptake studies *in vitro* Incubation of EGFR expressing **(A)** LS174T cells with polyplexes at a N/P ratio of 6 resulted in high transduction efficiency and EGFR-specificity of NIS-encoding LPEI-PEG-GE11/NIS (n=4) polyplexes compared to polyplexes without ligand (LPEI-PEG-Cys/NIS; n=4) and empty polymers (LPEI-PEG-GE11/HBG; n=4) **(B)**. Pre-treatment with the NIS-specific inhibitor perchlorate resulted in reduced iodide uptake confirming NIS-specificity. Cell viability was not affected by polyplex-mediated NIS gene transfer **(C)** (^*^p≤0.05; ^**^p≤0.01; ^***^ *p*≤0.001; n/s = not significant). Results are reported as mean ± SEM.

To evaluate optimal transfection conditions for complexes of LPEI-PEG-GE11 and NIS pDNA (LPEI-PEG-GE11/NIS polyplexes) in human CRC cells LS174T, which showed EGFR expression as determined by FACS analysis (Figure 1A), radioiodide uptake activity was evaluated 24 h after polyplex application. To obtain highest transfection rate with lowest impact on cell viability, a nitrogen/phosphate (N/P) ratio of 6 was chosen for all subsequent experiments. At 24 h after transfection with EGFR-targeted LPEI-PEG-GE11/NIS polyplexes, cells demonstrated a significantly higher uptake of ^125^I in comparison to cells that were incubated with LPEI-PEG-Cys/NIS, where the targeting ligand was replaced by cysteine. Additional pretreatment of cells transfected with LPEI-PEG-GE11/NIS with the NIS-specific inhibitor perchlorate (NaClO4) resulted in decreased uptake levels, comparable to the background levels measured after transfection with empty polymer in HBG buffer (LPEI-PEG-GE11/HBG) (Figure 1B). No effects on cell viability after transfections were detected (Figure 1C).

### ^18^F-TFB PET imaging after EGFR-targeted NIS gene delivery

**Figure 2:**
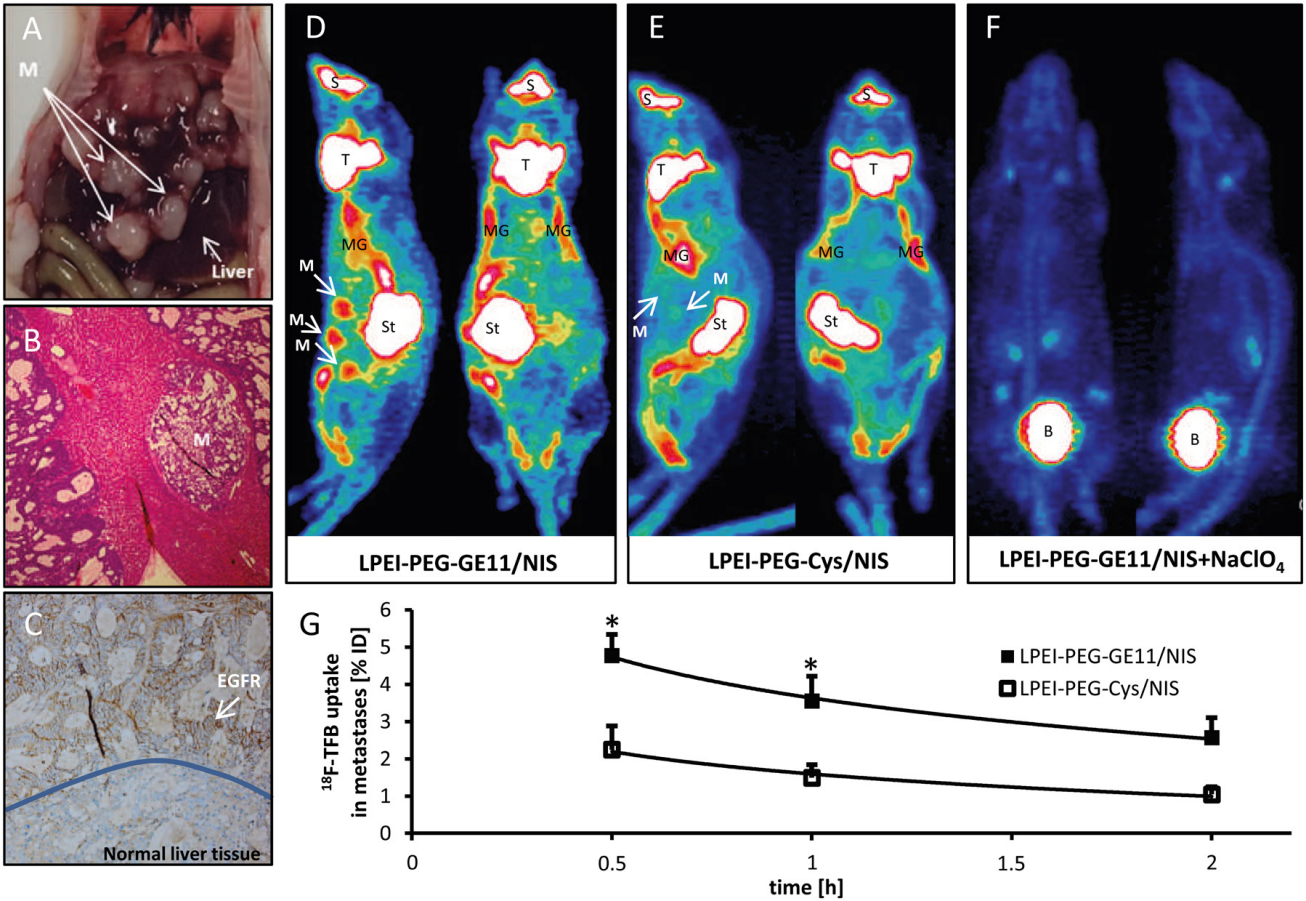
PET imaging studies after systemic NIS gene transfer *in vivo* Hepatic colon cancer metastases **(A, B)** showed high EGFR expression **(C)** in contrast to normal liver tissue. Significant tumor-specific accumulation of the NIS PET tracer ^18^F-TFB was detected in metastases of animals that received LPEI-PEG-GE11/NIS (**D**; n=5) in contrast to mice injected with control vectors (**E**; n=3). To verify NIS-mediated tracer uptake, LPEI-PEG-GE11/NIS-injected mice received the competitive NIS inhibitor perchlorate (NaClO4) 30 min before tracer administration (**F**; n=2), which blocked NIS-mediated tracer accumulation in metastases and tissues that exhibit endogenous NIS expression. Serial scanning revealed an accumulated dose of 4.8±0.6% of injected dose **(G)** (^*^p≤0.05). Results are reported as mean ± SEM. (M=metastases; S=nasal secretion; T=thyroid; MG=mammary gland; St=stomach; B=bladder).

Once animals had developed hepatic colon cancer metastases (Figure 2A and 2B), tumor sections were dissected and investigated for EGFR expression. High EGFR expression levels were detected in metastatic areas by immunohistochemical staining, whereas in normal liver tissue only low EGFR expression was observed (Figure 2C). Based on these results, animals with hepatic metastases received EGFR-targeted polyplexes LPEI-PEG-GE11/NIS or control polyplexes LPEI-PEG-Cys/NIS and functional NIS expression was imaged by 3-dimensional high resolution small animal PET 48 h after polyplex injection. High accumulation of the NIS PET tracer ^18^F-TFB was detected in metastases of animals that received LPEI-PEG-GE11/NIS polyplexes (Figure 2D). Serial scanning revealed an accumulated dose of 4.8±0.6 % of initial dose (ID) (Figure 2G). Non-targeted gene delivery via LPEI-PEG-Cys polymers resulted in a significantly reduced tumoral tracer uptake of 2.2±0.6 % of ID (Figure 2E and 2G). ^18^F-TFB uptake was also observed in physiologically NIS-expressing tissue (thyroid, mammary glands, salivary glands and stomach), as well as in the urinary bladder that is responsible for radionuclide elimination (Figure 2D and 2E). To verify that the tracer uptake in metastases was indeed NIS-mediated, LPEI-PEG-GE11/NIS-injected mice were additionally treated with the competitive NIS inhibitor perchlorate 30 min before tracer administration (Figure 2F). In these animals, NIS-mediated tracer accumulation was blocked in metastases and in tissues that exhibit endogenous NIS expression.

### *Ex vivo* analysis of NIS expression in CRC metastases

**Figure 3:**
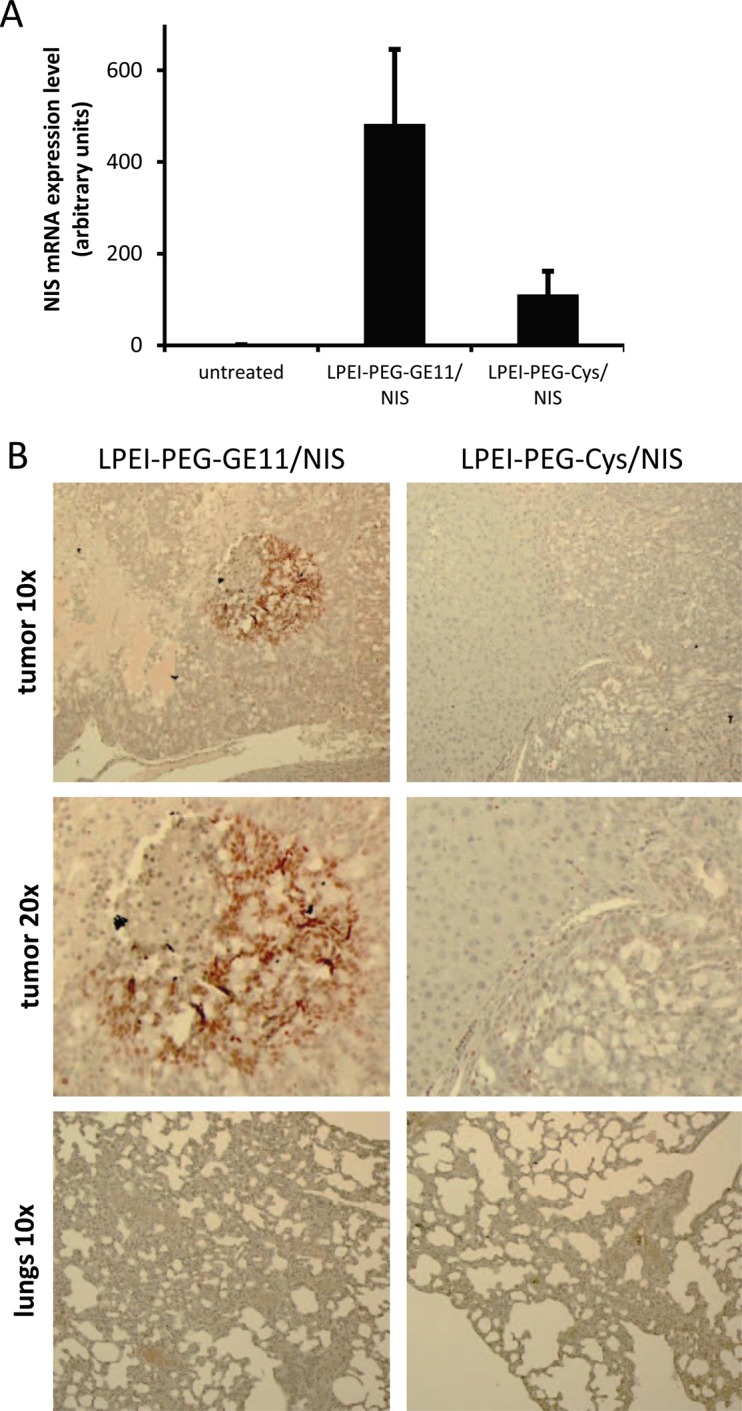
NIS expression in metastatic tumor sections qPCR revealed high NIS mRNA expression in metastatic areas of mice injected with LPEI-PEG-GE11/NIS (n=5) compared to untreated tumors (n=2) and low NIS mRNA expression levels were observed in tumors of mice that were treated with the control vector LPEI-PEG-Cys/NIS (n=3) **(A)**. Results are reported as mean ± SEM. Immunohistochemical staining confirmed NIS expression specifically in metastatic tissue of LPEI-PEG-GE11/NIS treated mice. NIS-specific immunostaining was visible only in metastatic tissue and occurred in clusters. No NIS expressing cells were found in normal liver tissue **(B)**.

To determine NIS mRNA expression in liver metastases by qPCR, mice were sacrificed 48 h after polyplex administration and tumors were dissected. High NIS mRNA expression was detected in metastatic areas in mice injected with LPEI-PEG-GE11/NIS as compared to untreated tumors. In addition, low NIS mRNA expression levels were observed in tumors of mice treated with the control vector LPEI-PEG-Cys/NIS (Figure 3A).

For further evaluation of NIS protein expression, tumor sections were stained with a human NIS-specific antibody. Immunohistochemical staining revealed areas of NIS-specific immunoreactivity in metastatic tissue of LPEI-PEG-GE11/NIS-treated mice, whereas surrounding normal liver and tumors from mice treated with the control vector LPEI-PEG-Cys/NIS showed no NIS-specific immunoreactivity (Figure 3B).

### NIS-mediated ^131^I therapy

**Figure 4:**
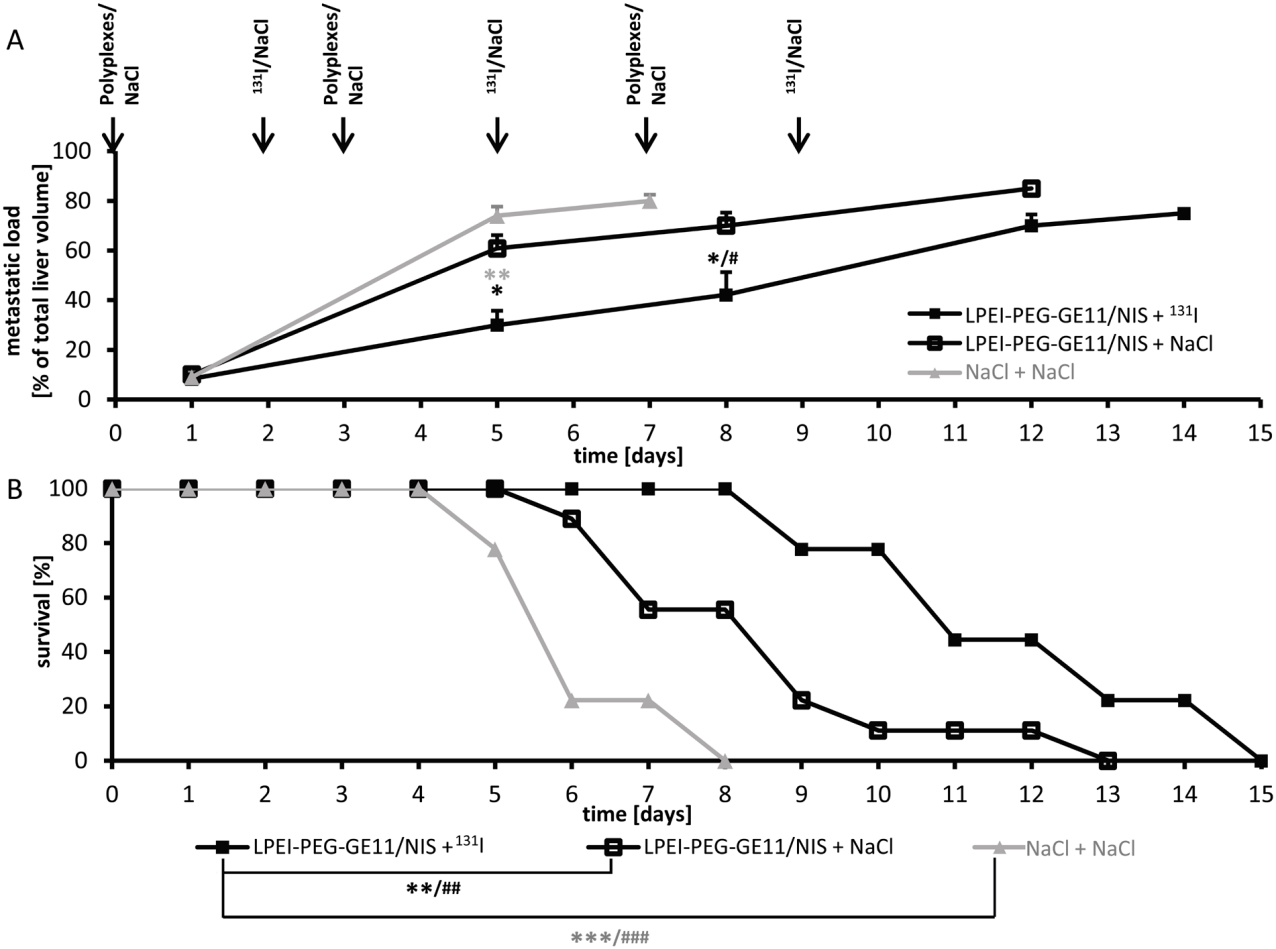
Radioiodide therapy studies Metastases-bearing mice were treated with three cycles of either LPEI-PEG-GE11/NIS followed by ^131^I (n=9) or saline (n=9) 48 h later or received saline only (n=9). Hepatic tumor load **(A)** and animal survival (Kaplan-Meier-Plot) **(B)** of the three different treatment groups were compared (Student's *t*-test: ^*^p≤0.05; ^**^p≤0.01; ^***^ *p*≤0.001; Mann-Whitney *U* test: **^#^***p*≤0.05; **^##^***p*≤0.01; **^###^***p*≤0.001). Results are either reported as mean ± SEM for tumor volumes or in percent for survival plots.

Metastases-bearing mice were treated with three cycles of either LPEI-PEG-GE11/NIS followed by ^131^I (therapy group) or saline 48 h later or received saline only. Mice in the therapy group showed a significant reduction of tumor growth reflected in hepatic metastases load as determined by contrast-enhanced ultrasound (CEUS), while aggressive tumor growth was observed in both control groups (Figure 4A). The reduced tumor growth in the therapy group resulted in an enhanced survival of these animals of up to 15 days post therapy start in contrast to control animals, that died within 8 days (NaCl + NaCl) group or 13 days (LPEI-PEG-GE11/NIS + NaCl group) (Figure 4B).

**Figure 5:**
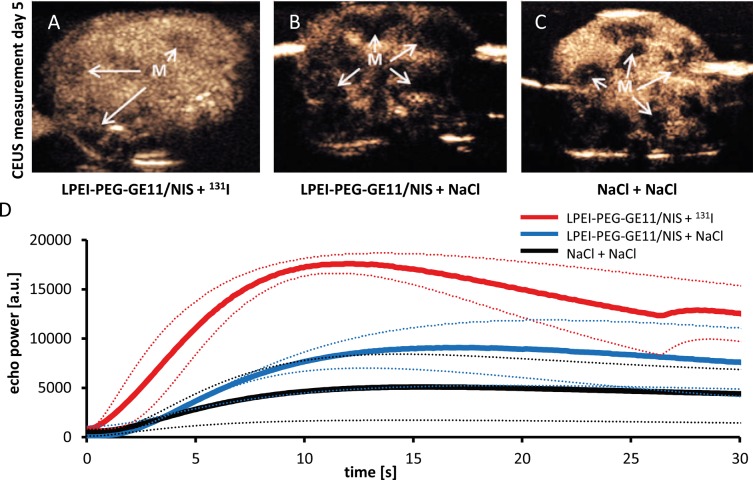
Hepatic contrast enhanced ultrasound (CEUS) measurement CEUS imaging of the entire liver on day 5 after therapy start revealed strong differences in contrast agent uptake and perfusion between therapy (**A**; LPEI-PEG-GE11/NIS + ^131^I) and control groups (**B**; LPEI-PEG-GE11/NIS + NaCl and **C**; NaCl + NaCl). Due to hypovascularization of metastases and necrotic areas metastases (M) appear dark. Animals treated with ^131^I showed an overall enhanced contrast agent signal and a higher maximum uptake **(D)**, in comparison to both control groups, which exhibited higher hepatic metastases load, reduced amount of healthy hepatic tissue and thus overall decreased perfusion levels.

For sonographic measurements 100 μl of the contrast agent SonoVue®, which consists of gas-filled microbubbles (diameter 2.5 μm), was intravenously applied. CEUS images showing the entire liver on day 5 after therapy start revealed a more homogenous uptake of contrast agent with only small areas with lower contrast agent uptake that represent metastases in animals that received LPEI-PEG-GE11/NIS + ^131^I (Figure 5A). In comparison both control groups, which exhibited higher hepatic metastases load with highly hypovascularized central areas, showed severely inhomogeneous contrast agent uptake (Figure 5B and 5C). To determine perfusion of disseminated liver metastases, echo signals were calculated by including the perfusion of the entire liver. A typical distribution of contrast agent in the LPEI-PEG-GE11/NIS + ^131^I group was detected, which showed a maximum peak of liver uptake 10 s after injection, followed by a quick tail off due to elimination. In the control groups no peak was observed. Regions of interest (ROI) were drawn around the entire liver to calculate and compare perfusion in the different treatment groups. While therapy animals that received LPEI-PEG-GE11/NIS + ^131^I revealed an increased overall signal, decreased amount of contrast agent uptake in the liver of control animals was detected (Figure 5D).

### Immunofluorescence staining for tumor cell proliferation and blood vessel density

**Figure 6:**
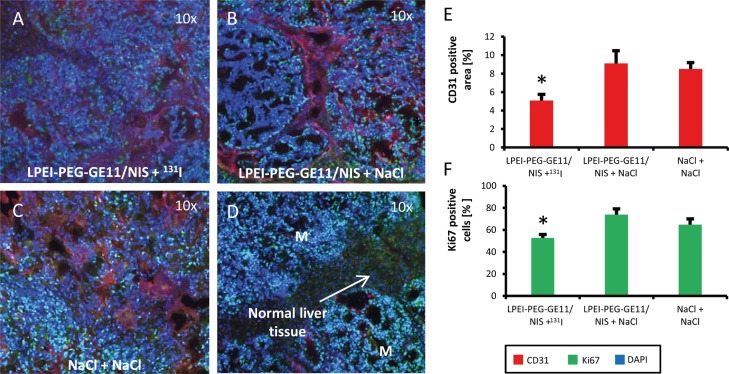
Immunofluorescence staining for tumor cell proliferation and blood vessel density Frozen metastatic tissue sections of the three different treatment groups exhibited reduced cell proliferation (green; Ki67) and blood vessel density (red; CD31) in animals treated with LPEI-PEG-GE11/NIS + ^131^I (n=9; A) compared to control groups that received LPEI-PEG-GE11/NIS + NaCl (n=9; B) or NaCl + NaCl (n=9; C). Differences in Ki67 and CD31 staining between metastatic tissue and normal liver tissue can be seen in **(D)**. Higher cell proliferation and increased pattern of vascularization of tissue surrounding metastases along with low vascularization inside metastases was found in both control groups **(E, F)** (^*^p≤0.05). Results are reported as mean ± SEM.

Metastatic liver tissue of mice after therapy was examined for cell proliferation with a Ki67-specific antibody (green) and for blood vessel density with a CD31-specific antibody (red) (Figure 6A-6C). Ki67 and CD31 staining in metastatic tissue differs from staining of normal liver tissue (Figure 6D). Metastatic tissue of LPEI-PEG-GE11/NIS-treated mice that received ^131^I showed significantly decreased proliferation and lower blood vessel density as compared to control groups (Figure 6E and 6F).

## DISCUSSION

In the emerging field of theranostics, which combine the capacities to function as imaging tool as well as a therapeutic agent, NIS has been evaluated as promising and beneficial target for cancer gene therapy. Due to its iodide concentrating ability, NIS expression in nonthyroidal tumor cells induced by targeted gene transfer enables non-invasive tumor monitoring and exact localization of the tumorous tissue by various imaging modalities (y-camera, PET, SPECT) and tracers (^123^I, ^188^Re, ^124^I, ^18^F-TFB, ^18^F-SO_4_) before therapeutic radionuclide application (^131^I, ^188^Re, ^211^At). The dual mechanism of NIS has been investigated in several nonthyroidal tumor models [3, 11–17, 19–21, 25, 30–34] and the concept of NIS gene therapy is currently under clinical evaluation for local treatment of prostate cancer after intratumoral application of adenoviral NIS gene carriers (NCT00788307, NCT01846091, NCT02068794) [32, 35]. Based on these results, the powerful image-based cytoreductive NIS gene therapy approach may represent a promising cancer treatment strategy even for metastatic disease.

However, a worthwhile evaluation of novel therapeutics for clinical use against metastases remains challenging. Thus, tumor models that give a detailed reflection of the natural processes in tumorigenesis, invasion and angiogenesis are highly important, especially for metastases formation, which is defined by a multi-step process. This process involves cell migration of tumor cells via blood vessels and lymphatic vessels to distant sites followed by generation of one or more micro-metastases that transform to macroscopic metastases with a unique microenvironment that is formed of the tumor cells themselves, tumor stroma and tumor-associated cells [36].

With respect to the high incidence of CRC as third most common cancer worldwide [2], which lacks new therapeutic options with clinical benefits, improved response and survival, especially for metastatic CRC where only a low percentage of patients are eligible for surgery, we established an *in vivo* metastases colon cancer model to investigate the NIS gene therapy concept as a new treatment option for CRC metastases. The CRC cell line LS174T, which was well characterized *in vitro* and showed promising results after NIS gene transfections, was injected directly into the mouse spleen, which results in tumor cell migration via the splenic vein and portal vein to the liver with cell invasion into the liver followed by formation of small metastases. After three days a splenectomy was performed to eradicate interference from a primary tumor in the spleen. With about 90% of mice developing liver metastases, this model is highly reproducible and metastases in other tissues, e.g. in the lungs were not observed.

To obtain effective NIS gene expression specifically in metastases, potent carrier systems are required. Various vector systems for systemic NIS gene transport have been investigated, including mesenchymal stem cells, viral and nonviral vehicles [3, 11–17, 19–21, 30]. Nonviral vectors as part of the nanotechnology field showed promising features for a targeted delivery to tumor tissue due to the variable design strategies, which allow adjustment on efficacy, biocompatibility and safety conditions [28]. Since the epidermal growth factor receptor (EGFR) is overexpressed in a high range of CRC tumors [37, 38], it represents an auspicious target for tumor-specific NIS gene delivery via actively targeted nanoparticle transfer. Based on promising earlier *in vivo* results [15, 29], for NIS gene transfer to metastatic CRC tissue LPEI-PEG-based polymers coupled to the EGFR-specific ligand GE11 were applied. LPEI as nonviral gene shuttle vector demonstrates high DNA binding capacity along with high transduction efficiency *in vitro* and *in vivo* and has already been evaluated in human clinical studies (NCT00712530) [39]. The additional PEG-shielding domain enables higher colloidal stability after systemic application and allows a safer *in vivo* application by reducing interactions with serum proteins and aggregation.

*In vitro* transfection studies in CRC LS174T cells using EGFR-targeted LPEI-PEG-GE11/NIS polyplexes resulted in significant perchlorate-sensitive ^125^I accumulation, whereas transfections with untargeted polyplexes showed only low iodide uptake above background levels. These results confirm the advantage of EGFR-targeting compared to the Cys control that lacks a specific cell-binding structure. This has already been demonstrated in former studies using various high EGFR expressing cell lines such as HuH7 liver cancer cells and SKOV-3 ovarian carcinoma cells [15]. Perchlorate sensitivity of iodide uptake in LPEI-PEG-GE11/NIS transfected cells confirmed NIS-mediated uptake.

Liver sections with metastases derived from intrasplenic injection of LS174T cells where stained for EGFR. High expression levels could be detected in the metastatic tissue providing the basis for EGFR-targeted NIS gene transfer *in vivo*. To be able to see NIS expression in single metastases and thereby confirming the suitability of our EGFR-targeted vectors for *in vivo* NIS gene delivery, we applied the novel NIS PET tracer ^18^F-TFB. Due to the small size of metastases with a diameter of around 1-5 mm, a precise evaluation for exact quantitative calculations of radionuclide uptake was required. Compared to the drawbacks of ^124^I as tracer with its long half-life, low positron yield, high positron energy, high energy gamma emission, limited availability and complex and expensive production, the new NIS PET tracer ^18^F-TFB promises a higher resolution and more differentiated images [40]. TFB was identified to be a substrate for NIS and by coupling ^18^F to TFB an ideal PET tracer with a short half-life and higher resolution images was developed [40]. Once mice had developed metastases, 2-3 weeks after tumor cell injection, animals were injected with LPEI-PEG-GE11/NIS or LPEI-PEG-Cys/NIS at a N/P ratio of 6. At 48 h after polyplex administration, mice received an intraperitoneal dose of 10 MBq ^18^F-TFB. To demonstrate that tracer uptake was indeed NIS dependent, a subgroup of the LPEI-PEG-GE11/NIS mice was injected with the NIS specific inhibitor perchlorate 30 min prior to ^18^F-TFB application. Whereas metastases could nicely be detected in the LPEI-PEG-GE11/NIS group due to strong NIS-mediated ^18^F-TFB accumulation, only weak radionuclide uptake was detected in metastases of the control group.

After this first series of encouraging animal imaging experiments, which confirmed high NIS expression in single metastases, the therapeutic efficacy of this approach was investigated. Animals received three cycles of polyplexes followed by ^131^I or saline application 48 h later or received saline only. Hepatic metastases load was monitored by conventional sonography and CEUS performed by an experienced radiologist in a blinded, randomized trial. Significantly reduced hepatic metastases load was detected in the LPEI-PEG-GE11/NIS group that received ^131^I, which was associated with prolonged survival, compared to both control groups.

For a detailed evaluation of the therapeutic effect, contrast agent distribution and liver perfusion were determined. As a consequence of the strong dissemination of small metastases pervading the entire liver, the evaluation of single metastasis was not feasible. Consequently, regions of interest (ROI) were drawn around the entire liver to calculate and compare perfusion in the different treatment groups.

For sonographic measurements 100 μl of the contrast agent SonoVue®, which consists of gas-filled microbubbles (diameter 2.5 μm), was intravenously applied. The process of contrast agent distribution in the liver consists of two dynamic phases. First, the arterial phase, which starts a few seconds after injection of the contrast agent, followed by the portal phase, where an overall enhancement of perfusion of normal liver tissue takes place [41]. The typical distribution of contrast agent results in a maximum peak of liver uptake and is followed by a quick elimination by respiration and elimination by the liver [42]. The majority of liver metastases derived from colorectal cancer is hypovascularized and shows contrast agent accumulation only at the periphery of the lesion [41, 43]. This pattern of increased vascularization of the tissue surrounding metastases along with low vascularization inside metastases could be confirmed by immunofluorescence analysis of vascularization (CD31-staining). Moreover, the extensive spread of the LS174T derived metastases in control mice lead to highly necrotic areas in the center of metastases, further diminishing vascularization inside metastases [44, 45].

Due to this hypovascularization of metastases together with a lower fractional vascular volume of metastases compared to normal liver tissue, absence of portal supply and a greater extent of necrotic areas, metastases generally appear dark [43, 46]. Hence, the livers of therapy animals that were injected with LPEI-PEG-GE11/NIS + ^131^I, where a reduced hepatic metastases load was detected, exhibited a more homogenous contrast agent uptake with only small areas that appear dark and represent metastases. In contrast, both control groups showed strongly inhomogeneous agent distribution and a high extent of metastases pervading the livers. Owing to the higher hepatic metastases load and thus reduced amount of healthy hepatic tissue where contrast agent uptake and perfusion can be detected, this resulted in overall decreased perfusion levels of control animals.

As hypovascularization often constitutes a major drawback for efficient delivery, in this study an active EGFR targeting approach was used. Hereby, gene delivery is not only dependent on the enhanced permeability and retention effect (EPR), where hypervascularization is an essential prerequisite for effective accumulation of vectors at the target site [47]. Efficacy of the active EGFR targeting strategy was proven by imaging and therapy studies. Further, different sizes of polyplexes and contrast agent particles may affect liver distribution. Sizes of polyplexes were detected to have an average size of around 100 nm, whereas particles of the contrast agent are specified to be at 2.5 μm. Thus, polyplexes are able to reach even small blood vessels.

In addition, the bystander effect that is associated with radioiodide therapy supports an effective treatment of non-transfected tumor cells in the surrounding tumor tissue due to the crossfire effect of the beta-emitter ^131^I of up to 2.4 mm. Hence, areas with diminished blood vessel density benefit from this bystander effect and can be efficiently destroyed even though they do not exhibit sufficient vascularization.

In conclusion, our data clearly demonstrate the potential of LPEI-PEG-GE11 carrier systems to target the NIS gene to hepatic CRC liver metastases. NIS as reporter gene allows for quantification of the extent of gene expression and quantitative analysis of tracer uptake. In its function as therapeutic gene, after applying the therapeutic tracer ^131^I, NIS-mediated ^131^I accumulation induces decelerated metastatic tumor growth with prolonged animal survival. The established metastases model is a valuable tool to reflect the clinical situation on a morphological and molecular level and serves as an ideal advanced tumor model to investigate our EGFR-targeted NIS-mediated gene therapy approach.

## MATERIALS AND METHODS

### Cell culture

The human colon carcinoma cell line LS174T (ATCC-CCL188; American Type Culture Collection, Manassas, VA) was cultured in Eagle's Minimum Essential Medium (EMEM; Sigma-Aldrich, St.Louis, MO) supplemented with 10% (v/v) fetal bovine serum (FBS Superior, Biochrom/Merck Millipore, Berlin, Germany), 5% (v/v) L-glutamine (Sigma-Aldrich) and 1% (v/v) penicillin/streptomycin (Sigma-Aldrich). Cells were maintained at 37°C and 5% CO_2_ in an incubator with 95% humidity. Cell culture medium was replaced every second day and cells were passaged at 80% confluency.

### Plasmid and polymer synthesis and polyplex formation

Polymers with LPEI-PEG backbone (EGFR-targeted: LPEI-PEG-GE11; control polymer without ligand: LPEI-PEG-Cys) and codon-optimized human NIS cDNA (NIS pDNA) were synthesized as described previously [15, 30]. Polymers and cDNA were diluted in same volumes of HEPES (2-[4-(2-hydroxyethyl) piperazin-1-yl]ethanesulfonic acid)-buffered glucose (HBG: 20 mmol/l HEPES, 5% (w/v) glucose at pH 7.4) at a N/P ratio of 6 (w/w). Polymers were added to the DNA by rapid mixing and incubated at room temperature for 20 min prior use [48]. For *in vitro* studies the concentration of DNA for polyplex formation was 2 μg/ml, for *in vivo* studies 200 μg/ml.

### EGFR expression levels *in vitro*

1 × 10^6^ LS174T cells were detached with trypsin, centrifuged and incubated with an EGFR-specific antibody (1:100; monoclonal mouse IgG1 - Dako, Glostrup, Denmark) or with an IgG-anti-mouse antibody (BD Bioscience, Franklin Lakes, USA) as negative control in FACS buffer (PBS with 10% FBS) for 1 h on ice. Cells were washed with FACS buffer and incubated with an AlexaFluor 488 labeled goat anti-mouse secondary antibody (1:400 - Invitrogen, Langenselbold, Germany) for 1 h on ice. After washing, cells were resuspended in FACS buffer and flow cytometry analysis was performed on a BD Accuri C6 flow cytometer (BD Bioscience, Franklin Lakes, USA). Cells were gated by forward/sideward scatter and pulse width for exclusion of doublets. PI (propidium iodide, Sigma-Aldrich) was used for discrimination between viable and dead cells.

### Transfection studies

CRC cells LS174T were plated at a density of 1×10^6^ cells per well, grown to 60 - 80% confluency in 6-well plates and incubated for 24 h with either LPEI-PEG-GE11/NIS, LPEI-PEG-Cys/NIS or empty polymer LPEI-PEG-GE11/HBG. Transfection efficiency was determined by measurement of iodide uptake activity at steady-state conditions as described previously [25]. Results were normalized to cell survival measured by cell viability assay (see below) and expressed as cpm/A620.

### Cell viability assay

Cell viability after transfection was analyzed after incubation of cells with a commercially available MTT reagent (Sigma-Aldrich) for 2 h at 37°C followed by a washing step with PBS (phosphate-buffered saline). The formazan product was measured after incubation with 10% DMSO (v/v) (dimethyl sulfoxide) in isopropanol at 620 nm in a Sunrise microplate absorbance reader (Tecan, Männedorf, Switzerland).

### Establishment of hepatic colorectal metastases in nude mice

For the establishment of CRC metastases in female CD-1 nu/nu mice (Charles River, Sulzfeld, Germany), animals were fully anesthetized and after laparotomy, 50 μl of tumor cell suspension (1×10^6^ cells in 1×PBS) was injected into the upper splenic pole. After 3 days, the abdominal wall was re-opened at the same site and a splenectomy was performed. Mice were pre- and post-treated with Metacam (0.5 mg/kg) to minimize wound pain and reduce risk of inflammation. 2-3 weeks after intrasplenic injection mice bore multifocal liver metastases. Mice were sacrificed when healthy liver tissue reached less than 30%, in case of weight loss of more than 10% of initial weight, or when impairment of breathing, drinking or eating behavior was observed. Animals were maintained under specific pathogen-free conditions with access to mouse chow and water *ad libitum*. The experimental protocol was approved by the regional governmental commission for animals (Regierung von Oberbayern) and all animal experiments were carried out according to the guidelines of the German law of protection of animal life.

### EGFR-immunohistochemistry staining

Immunohistochemistry staining of EGFR on paraffin-embedded tumor tissue was performed as described previously [49].

### PET imaging studies after systemic NIS gene transfer *in vivo*

Experiments started 2-3 weeks after intrasplenic injection of tumor cells. To demonstrate tumor-specific NIS expression *in vivo*, mice received polyplexes systemically via the tail vein (i.v.) at a DNA dose of 2.5 mg/kg (50 μg DNA in 250 μL HBG). Mice were treated as follows: (1) LPEI-PEG-GE11/NIS (n=5); (2) LPEI-PEG-Cys/NIS (n=3); (3) LPEI-PEG-GE11/NIS + NaClO_4_ (n=2): pretreatment with an i.p. injection of 2 mg of the competitive NIS inhibitor sodium perchlorate (NaClO_4_) 30 min before PET tracer administration. 48 h after polyplex injection the novel NIS PET tracer ^18^F-TFB (10 MBq; synthesized by the Radiopharmacy, Klinikum rechts der Isar, TU Munich, as described previously [40]) was applied i.v. and tracer accumulation in metastatic liver areas was determined by small-animal PET (Inveon, SIEMENS Preclinical Solutions, Erlangen, Germany). Regions of interest were analyzed with the software Inveon Acquisition Workplace (Siemens) and quantified using Inveon Research Workplace (Siemens) and expressed as a fraction of the total amount of initial dose (% of ID). The retention time of the tracer ^18^F-TFB within metastases was determined by serial scanning after 0.5 h, 1 h and 2 h. To suppress thyroidal iodide uptake, a 10-day pretreatment with L-thyroxin (L-T4; 5 mg/ml; Sigma-Aldrich) in drinking water was conducted before PET-imaging.

### Analysis of NIS mRNA expression by quantitative real-time PCR

For quantification of NIS expression in metastatic tissue, total RNA was isolated using the RNeasy Mini Kit (Qiagen, Hilden, Germany) according to the manufacturer's recommendations. Reverse transcription was performed using SuperScript III First-Strand Synthesis System (Thermo Fisher Scientific, Waltham, Massachusetts, USA). Quantitative real-time PCR was run with the QuantiTect SYBR Green PCR Kit (Qiagen) in a Mastercycler ep gradient S PCR cycler (Eppendorf, Hamburg, Germany). Relative expression levels were calculated from ΔΔCt values normalized to internal β-actin. As primers, the following sequences were used:

*hNIS*: (5′ACACCTTCTGGACCTTCGTG-3′) and (5′-GTCGCAGTCGGTGTAGAACA-3′),

*β-actin*: (5′AGAAAATCTGGCACCACACC-3′) and (5′-TAGCACAGCCTGGATAGCAA-3′).

### Immunohistochemistry analysis of NIS protein expression

Immunohistochemical staining of paraffin embedded tumor tissue derived from hepatic metastases sections after NIS gene delivery were performed using a mouse monoclonal antibody directed against human NIS (kindly provided by John C Morris, Mayo Clinic, Rochester, MN, USA) as described previously [19, 50]. Immunohistochemically stained sections were imaged on an Olympus BX41 microscope equipped with an Olympus XC30 CCD camera (Olympus, Shimjukum Tokio, Japan).

### Radioiodide therapy studies

Therapy trials were started two weeks after intrasplenic injection, when mice showed low hepatic metastases load (<20%), which was determined by CEUS. To this end, animals were injected i.v. with either LPEI-PEG-GE11/NIS followed by an i.p. application of 55.5 MBq ^131^I or saline 48 h later or received saline only (LPEI-PEG-GE11/NIS + ^131^I (n=9); LPEI-PEG-GE11/NIS + NaCl (n=9); NaCl + NaCl (n=9)). The cycle consisting of systemic NIS gene transfer followed by radioiodide was repeated for a total of three times on days 0/2, 3/5 and 7/9.

Hepatic colorectal metastases load was monitored by CEUS as described by Eichhorn *et al*. [51]. CEUS was performed on an Acuson Sequoia 512 (Siemens) in combination with a 15L8W ultrasound probe using the Cadence contrast pulse sequencing technology. Metastases were imaged in brightness-mode with a frequency of 14 MHz and a mechanical index of 0.2 or less. The contrast agent SonoVue® (Bracco, Milano, Italy) was applied via a tail vein catheter. Hepatic contrast agent distribution and accumulation in normal liver tissue and metastases was recorded on digital cine clips before and up to 30 s after the application of the contrast agent at a frame rate of 8-10 Hz. Digital cine clips were exported in a Digital Imaging and Communications in Medicine (DICOM) format for off-line analysis with VueBox (Bracco Suisse, Geneve, Switzerland) using a bolus kinetic model as described previously [3]. In a blinded experimental set-up, hepatic metastases load was estimated by an experienced radiologist with more than 16 years’ experience in CEUS measurement using high-end ultrasound devices. For estimation of perfusion and contrast agent uptake, region of interests were drawn around the entire liver of each animal. The contrast agent concentration was estimated using pre-defined calibration curves [51].

Two independent therapy trials were conducted. For the first trial, the follow-up CEUS measurements were performed on days 8 and 12 after therapy start, in the second therapy trial a single follow-up CEUS measurement was performed on day 5. For evaluation of sonographic parameters, only animals from the second therapy trial with CEUS measurement on day 5, where clips were analyzable due to hepatic in-flow of the contrast agent, were included in calculations to enable a parallel comparison of all three treatment groups (LPEI-PEG-GE11/NIS + ^131^I (n=2); LPEI-PEG-GE11/NIS + NaCl (n=2); NaCl + NaCl (n=3)).

### Indirect immunofluorescence assay

Immunofluorescence staining was performed on dissected frozen tumor tissues as described previously [30]. A rabbit polyclonal antibody against human Ki67 (Abcam, Cambridge, UK; dilution 1:1000) and a rat monoclonal antibody against mouse CD31 (BD Pharmingen, Heidelberg, Germany; dilution 1:200) were used. For detection, an anti-rabbit Alexa488-conjugated secondary antibody (Jackson ImmunoResearch, West Grove, Pennsylvania, USA) for Ki67 staining and an anti-rat Cy3-conjugated secondary antibody (Jackson ImmunoResearch) for CD31 staining were applied. Nuclei were counterstained with Hoechst bisbenzimide (5 mg/ml) and sections were embedded in Fluorescence Mounting Medium (Dako, Hamburg, Germany). ImageJ software (NIH) was used for quantification of proliferation (Ki67-staining) and blood vessel density (CD31-staining) by analyzing 6 visual fields per metastatic liver section of every mouse.

### Statistics

All *in vitro* experiments were carried out at least in triplicates. Results are expressed as mean ± SEM, mean fold change ± SEM and, for survival plots, in percent. Statistical significance was calculated by two-tailed Student's *t*-test. *P* values ≤0.05 were considered significant (Student's *t*-test: ^*^p≤0.05; ^**^p≤0.01; ^***^p≤0.001). For therapy studies, in addition, Mann-Whitney *U* test was performed. *P* values ≤0.05 were considered significant (Mann-Whitney *U* test: **^#^***p*≤0.05; **^##^***p*≤0.01; **^###^***p*≤0.001).
